# Incorrect handling of calibration information in divergence time inference: an example from volcanic islands

**DOI:** 10.1002/ece3.94

**Published:** 2012-03

**Authors:** Beatriz Mello, Carlos G Schrago

**Affiliations:** Department of Genetics, Federal University of Rio de Janeiro,Rio de Janeiro, Brazil

**Keywords:** Bayesian-relaxed molecular clock, biogeography, Hawaii

## Abstract

Divergence time studies rely on calibration information from several sources. The age of volcanic islands is one of the standard references to obtain chronological data to estimate the absolute times of lineage diversifications. This strategy assumes that cladogenesis is necessarily associated with island formation, and punctual calibrations are commonly used to date the splits of endemic island species. Here, we re-examined three studies that inferred divergence times for different Hawaiian lineages assuming fixed calibration points. We show that, by permitting probabilistic calibrations, some divergences are estimated to be significantly younger or older than the age of the island formation, thus yielding distinct ecological scenarios for the speciation process. The results highlight the importance of using calibration information correctly, as well as the possibility of incorporating volcanic island studies into a formal, biogeographical hypothesis-testing framework.

## Introduction

Species divergence time inference has received a great deal of attention over the last decade following the development of methods based on Bayesian statistics (Bromham and Penny 2003). These new methods have eliminated the need for a strict molecular clock with a single homogeneous rate of molecular evolution to estimate absolute times (Thorne and Kishino 2002; Drummond et al. 2006). The methodological flexibility of the Bayesian framework also allows for the incorporation of calibration information in the form of probability distributions (Drummond et al. 2006; Rannala and Yang 2007), making chronological inference much more attractive because it naturally incorporates the uncertainty of the fossil record into the analysis.

Another commonly used source for obtaining calibration information is the geological record, especially events such as the formation of islands. In fact, the age of volcanic islands has been used extensively to obtain calibration information to estimate cladogenetic times in phylogeographic studies (Russo et al. 1995; Fleischer et al. 1998). However, this method comes with two major assumptions: (i) the age of the islands is known with a minimum error, and (ii) the lineages diverged at the moment the younger island was formed. This means that the speciation process cannot happen before or after that geological event (Fleischer et al. 1998; Heads 2011). Unfortunately, the majority of the studies conducted so far do not scrutinize their data according to these premises (Hormiga et al. 2003; Bonacum et al. 2005) and arbitrarily assume that island colonization occurred immediately after the formation of the island.

A critique of these assumptions was recently penned byHeads (2011), who reviewed studies that estimated divergence times that inadvertently adopted the aforementioned assumptions. It is clear that, despite the several modeling advances that have been implemented, the estimation of divergence times is heavily dependent on the quality of the calibration information (Ho and Phillips 2009). Nevertheless, the influence of the calibration priors on the posterior estimates of divergence times is rarely explored.

In this study, we re-examined recent works that estimated the divergence times of lineages from the Hawaiian Islands. Those are the analyses performed byJordan et al. (2003), Hormiga et al. (2003), and Givnish et al. (2009), which incorporated information about the age of the islands as calibration points for splits between the Hawaiian lineages. We show that when probabilistic calibration information is considered, the hypothesis of divergence immediately following island formation is discarded in favor of younger or older divergences. Such results demonstrate the error caused by the improper treatment of calibration information, and they highlight the capability of Bayesian divergence time methods to reject phylogeographic hypotheses about lineage divergences in hot-spot archipelagos.

## Materials and Methods

### Sequences and alignment

To investigate how the divergence times of island species are influenced by calibration information, we re-examined the following studies: Hormiga et al. (2003), hereafter referred to as study A; Jordan et al. (2003), referred to as study B; and Givnish et al. (2009), referred to as study C.Hormiga et al. (2003) conducted a phylogenetic analysis of the Hawaiian spider genus *Orsonwelles*, a group comprising 13 single-island endemic species (Hormiga 2002).Jordan et al. (2003) estimated the phylogenetic relationships of the damselfly genus *Megalagrion*, in which most species are endemic to single islands of the Hawaii archipelago (Jordan et al. 2003).Givnish et al. (2009) presented a molecular phylogeny of Hawaiian lobeliads, the largest family of Hawaiian angiosperms (Wagner 1999). All of these studies employed divergence time methods that treated calibration information as punctual; that is, they applied fixed calibration points instead of probabilistic distributions.

The alignment fromJordan et al. (2003) was downloaded from TreeBASE (Piel et al. 2002), and the datasets used inHormiga et al. (2003) andGivnish et al. (2009) were downloaded from GenBank (for accession numbers, please refer to Supporting Information Table S1). In each dataset, if two or more sequences presented genetic distances <0.003, only one was kept in the analysis. For these two datasets, all of the protein-coding sequences were aligned in PRANK (Loeytynoja and Goldman 2009), whereas the ribosomal RNA genes were aligned in R-coffee (Wilm et al. 2008). For each study, the individual genes were concatenated into a single supermatrix in SeaView (Gouy et al. 2010). Missing or ambiguous data and gaps were not excluded.

## Evolutionary analyses

Maximum likelihood phylogenetic inference was conducted in PhyML 3 (Guindon et al. 2010) under the GTR + G6 evolutionary model using the option in which the tree topology is the best solution of the heuristic searches using the nearest neighbor interchange and subtree pruning and regrafting searches. The starting tree was built with the BIONJ algorithm (Gascuel 1997).

We performed two independent analyses to measure the impact of the punctual calibration points on the divergence time inference. First, we relaxed the punctual chronological constraint on the calibrated nodes (as applied in previous studies) by assuming a normal prior distribution in which the mean was set at the same value as the punctual calibration of the original study (Table 1). Standard deviations were entered in order to allow the minimum 99% lower limit of the distribution to include zero (i.e., the present). Thus, we have permitted ample exploration of the chronological space.

**Table 1:** Calibration information used in each study.

Study	Normal prior (Mean ± SD)	Punctual prior [lower, upper]	Nodes	Geological reference
Hormiga et al. 2003 (*Orsonwelles*)	2.6 ± 1.1	[2.59, 2.61]	A	Oahu
Jordan et al. 2003 (*Megalagrion*)	3.7 ± 1.6	[3.69, 3.71]	B4, B7, and B9	Oahu
	1.6 ± 0.7	[1.59, 1.61]	B2, B3, B5, B6, and B8	Maui Nui
	0.5 ± 0.2	[0.49, 0.51]	B1	Hawaii
Givnish et al. 2009 (Hawaiian lobeliads)	5.2 ± 2.2	[5.19, 5.21]	C7	Niihau
	4.7 ± 2.0	[4.69, 4.71]	C1, C4, C5, C6, C8, and C9	Kauai
	3.0 ± 1.3	[2.99, 3.01]	C3	Oahu
	0.6 ± 0.3	[0.59, 0.61]	C2	Hawaii

**Figure 1:**
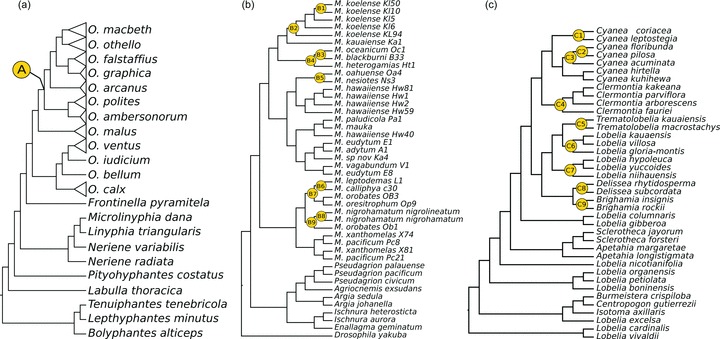
Maximum likelihood phylogenies inferred, indicating the calibrated nodes.

The second divergence time analysis was conducted with prior settings that replicated the punctual calibration points originally used in studies A, B, and C. To achieve this, the nodes were calibrated using very narrow time intervals limited by hard bounds (i.e., the values outside the interval had zero probability;Table 1). The studies we re-analyzed assumed different ages for the same Hawaiian Islands. For instance, Hormiga et al. (2003) set the age of Oahu Island to 2.6 Mega annum (Ma), whereasJordan et al. (2003) set the age to 3.7 Ma. To make the results comparable, we maintained all of the ages originally used by the studies (Table 1). Maximum likelihood tree topologies and nodes with calibration information are displayed in Figure 1.

All of the inferences of species divergence times were conducted under the Bayesian framework, as implemented in the MCMCTREE package of PAML 4.4d (Yang 2007) and BEAST 1.6.2 (Drummond and Rambaut 2007). Both programs produced equivalent results. In MCMCTREE, posterior distributions were obtained via the Markov chain Monte Carlo algorithm using the approximate likelihood calculation to accelerate the process (dos Reis and Yang 2011). In every analysis, after an adjustable burn-in period, the Markov chains were sampled every 100th cycle until 10,000 trees were obtained. Two independent replicates were performed to check for the convergence of the estimates. The effective sample sizes (ESSs) of the inferred parameters were calculated in Tracer v. 1.5 (Rambaut and Drummond 2007). Chains with a parametric ESS <200 were rerun until 200 was reached. The divergence between the normal prior distributions and the inferred posterior distributions of the calibrated nodes was measured using the Kullback–Leibler divergence (KLdiv; Kullback and Leibler 1951). We compared the divergence time inferences obtained from the two analyses by calculating the difference between the means of the posterior distributions of the ages of each node.

## Results

**Figure 2:**
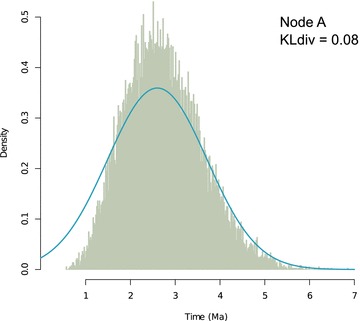
Prior (solid blue line) and posterior distributions of the age of the calibrated node used inHormiga et al. (2003). The value of the Kullback-Leibler divergence is also shown.

In several nodes, the posterior distributions were significantly different from the normal priors that we adopted. In study A, the only calibration used was the age of Oahu Island, here implemented as a normal distribution with a mean of 2.6 Ma and a standard deviation of 1.1 Ma. The posterior distribution for this split was very similar to the normal distribution used as prior to calibrate this divergence (Fig. 2), and the KLdiv value was low (0.08), indicating that this split may have occurred around the time of Oahu Island's formation. Thus, the hypothesis of rapid colonization after island formation cannot be rejected.

**Figure 3:**
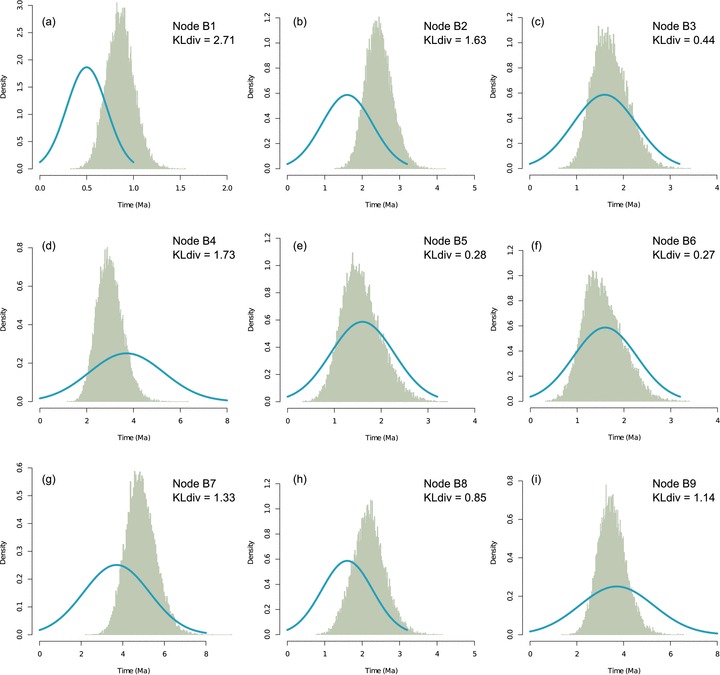
Prior (solid blue line) and posterior distributions of the age of the calibrated nodes used inJordan et al. (2003). The value of the Kullback–Leibler divergence is also shown.

In study B, three distinct normal priors were used as calibrations for nine nodes (B1–B9; Fig. 3). The posterior distributions for the ages of nodes B1, B2, B7, and B8 indicated a split earlier than the age of the islands represented by the prior. For instance, the 95% highest probability density (HPD) interval of the age of node B1 ranges from 0.6 to 1.1 Ma, which excludes the age of Hawaii Island (0.5 Ma). Moreover, the inferred KLdiv was the highest in study B (2.71). Therefore, the hypothesis of cladogenesis caused by island formation is rejected because the species were already diversified. In the case of nodes B2 and B8, the mean ages were older than Maui Nui (1.6 Ma). This was also true for node B7, which had a mean divergence time older than Oahu Island (3.7 Ma). However, the 95% HPD intervals of nodes B2, B7, and B8 did include the ages of the respective islands from the original studies; moreover, the KLdiv values were not high (1.63, 1.33, and 0.85, respectively). Thus, the hypothesis that species diversification is indeed influenced by island formation cannot be strictly rejected. However, the inferred age of node B4 points to a later divergence. The mean of the posterior distribution was estimated at 3 Ma, whereas the age of Oahu Island was set to 3.7 Ma. Nevertheless, the 95% HPD interval included the age of Oahu.

In all of the other posterior distributions (B3, B5, B6, and B9), the estimated means were very similar to the normal prior means. Therefore, one cannot reject the formation of the island as the causative event for the split. For the B3, B5, and B6 nodes, the KLdiv values were below 0.5. For node B9, the KLdiv value was relatively high, but this is because the shape of the posterior distribution was narrower around the mean.

**Figure 4:**
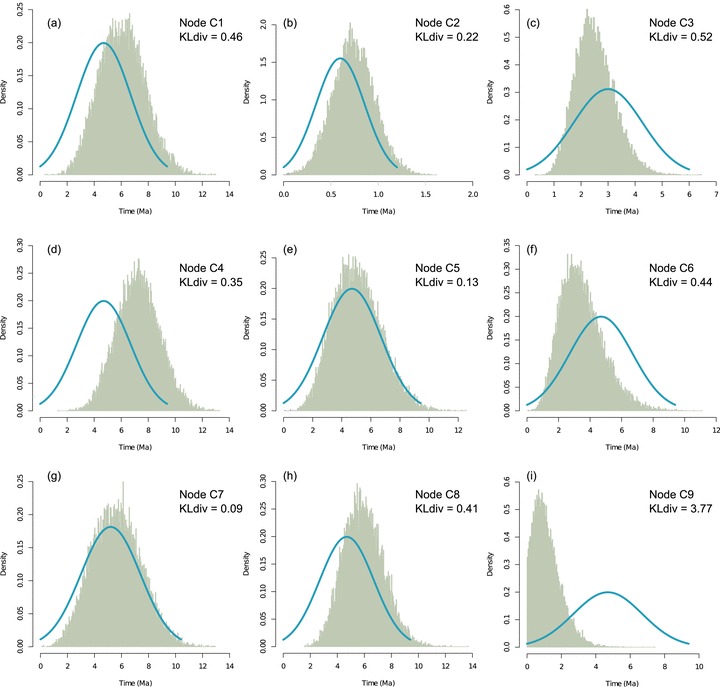
Prior (solid blue line) and posterior distributions of the age of the calibrated nodes used inGivnish et al. (2009). The value of the Kullback–Leibler divergence is also shown.

In study C, the divergence times of nodes C1, C2, C4, and C8 were inferred to be older than the age of Kauai (C1, C4, and C8) and Hawaii (C2) Islands (Fig. 4). The 95% HPD intervals, however, contained the mean age of the islands. Therefore, one cannot reject the hypothesis that cladogenetic events were influenced by island formation. This is further confirmed by the low values of the KLdiv, which varied from 0.22 (C2) to 0.46 (C1). Nodes C3, C6, and C9 were dated at ages later than the formation of their islands. This is most obvious in node C9, where the credibility interval of the estimate ranged from 0.0 to 2.7 Ma, a time significantly later than the formation of Kauai Island. The KLdiv values for splits C3 and C6 were lower than 1, whereas the KLdiv for node C9 was the highest found in our analysis (3.77). Lastly, the posterior distributions of nodes C5 and C7 had posterior distributions that were indistinguishable from their respective normal priors, which supports the hypothesis that cladogenesis is associated with island formation.

**Figure 5:**
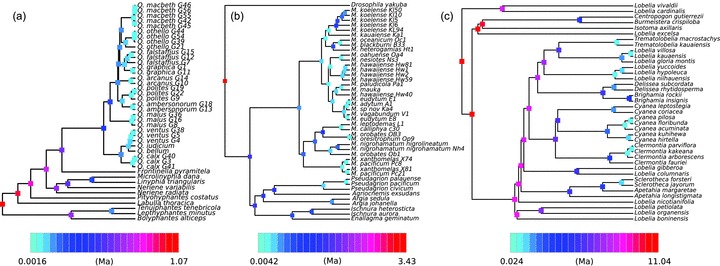
The difference between probabilistic and punctual calibrations. In each node, the magnitude of the difference is shown as represented in the scales below each tree.

When the estimates obtained by assuming the normal prior were compared with the punctual calibrations, it became evident that the influence of the probabilistic distributions was greater at the nodes located deeper in the phylogeny. The differences ranged from 0.0016 to 1.07 Ma in study A, 0.0042 to 3.43 Ma in study B, and 0.024 to 11.04 Ma in study C (Fig. 5).

## Discussion

By using a classic example of a geological formation that is widely applied as source of calibration points, we have emphasized the relevance of a general problem in divergence time estimation, namely, the incorrect assignment of geological or paleontological information to calibration priors. With respect to volcanic archipelagos, Heads (2011) recently criticized the use of island ages to date endemic island clades, emphasizing the problem of old taxa on young islands. For instance, the interpretations of Hawaiian biogeography are, according to this author, based on two assumptions: (i) “the intraplate volcanism on the Hawaiian Islands is well understood and caused by a fixed mantle plume hot spot” and (ii) “at one time in the Eocene-Oligocene none of the Hawaiian Islands (or no high islands) were emergent.”

Most biologists argue that Hawaiian geology is well understood (Cowie and Holland 2006), and they accept the traditional model of Hawaiian intraplate volcanism, where the movement of the Pacific plate over a fixed hotspot forms successively younger islands. However, intraplate volcanism, unlike volcanism on plate boundaries, is a field of intense debates among geologists (Heads 2011). Several key papers have discussed alternative interpretations of Hawaiian geology (Anderson 2005; Norton 2007; Sager 2007), and one of them points out that a number of key criteria for a hot-spot model are missing for Hawaii (Neall and Trewick 2008). Therefore, it is acceptable for there to be a degree of uncertainty associated with the ages of the Hawaiian Islands, which are summarized in a series of published works (Carson and Clague 1995; Price and Clague 2002).

Another important issue to be considered is that tree topologies must show that monophyletic taxa arose in parallel with the temporal ordering of the islands (Fleischer et al. 1998). If this is true, then the island ages for the Hawaiian archipelago can be employed as calibration points. However, some studies find different patterns, so the “progression rule” does not apply (Arnedo and Gillespie 2006; Holland and Cowie 2007). For instance, Holland and Cowie suggested that the basal clade of the amber snail, *Succinea caduca*, inhabits Hawaii Island, which is the youngest island in the chain. Arnedo and Gillespie did not find a pattern of taxa arising in the temporal order of the islands, and they attributed this to a possible “rapid dispersal through the islands by each lineage right after their origination,” suggesting that the *Havaika* spider genus arrived at the archipelago after Kauai, Oahu, and Maui Nui had already emerged. These uncertainties surrounding colonization patterns are a problem, especially when calibrations use fixed points without a probabilistic distribution associated with a node to account for uncertainty.

We demonstrated that the use of probabilistic calibration information for the ages of volcanic islands permits the divergence times of the nodes to be inferred at values younger or older than the age of the islands themselves. Such an approach, when compared to the punctual calibration, is statistically superior because it permits the formal biogeographical testing of the influence of geological events on cladogenesis (Crisp et al. 2011). Thus, such analysis should be considered a general tool for statistical analysis of biogeographical issues. The timescale of species diversification is also considerably affected, resulting in different interpretations of the scenarios in which clade evolution took place. In this sense, we found the greatest difference between our estimates and those ofGivnish et al. (2009). For instance, the authors estimated the age of the root of the Hawaiian-chain lobeliads to be approximately 13.6 ± 3.1 Ma. We inferred the same node to be 32.6 Ma (47.9–21.8). These estimates are statistically different. However, in the case ofHormiga et al. (2003) andJordan et al. (2003), the differences were not as significant. Hormiga et al. estimated the time of the most recent common ancestor of the *Orsonwelles* genus to be 4.5 Ma, whereas the same node was dated at 4.9 Ma in our analysis. Lastly, the age root of the *Megalagrion* diversification was inferred to have occurred 9.6 Ma byJordan et al. (2003) and 9.0 Ma in the present study.

In the case of Hormiga et al. (2003), similarities between the estimates were expected, as the posterior distribution of the calibrated node was indistinguishable from the normal prior. Despite the general agreement of our estimates with those ofJordan et al. (2003), it is worth mentioning that, because of the probabilistic framework adopted here, it was possible to reject the hypothesis of cladogenesis associated with the island formation at node B1 (Fig. 3). Thus, even if the timescales are comparable, our approach enabled us to perform an explicit biogeographical test.

It is difficult to demonstrate that the inability to reject the null hypothesis of concomitant species diversification and island formation necessarily means that speciation was caused by the geological appearance of the younger island. The power of the test is directly associated with the variance of the estimates and, consequently, with the information carried in the alignment, as measured by the likelihood function (Yang 2006). Thus, our approach to the historical biogeography of the Hawaiian Island chain is conservative because we only accept the scenario of younger or older diversification if the age of the island lies significantly outside the 95% HPD interval.

We believe that our method of analysis is statistically superior because, by avoiding punctual calibration information that expresses a priori certainty about the biogeographical process, we permit hypothesis testing of the association between the geological factors and lineage divergence. Such an approach differs from the classical cladistic biogeographical analysis (Nelson and Rosen 1981; Platnick and Nelson 1978; Rosen 1978), which is pattern based and ignores chronological information as a valid discriminatory argument (Donoghue and Moore 2003).

## Supporting Information

**Table S1**. GenBank accession numbers for the datasets usedin Hormiga et al. (2003), Jordan et al. (2003) and Givnish et al.(2009).
