# Supplementary material for: Incorrect handling of calibration information in divergence time inference: an example from volcanic islands
Source: Ecol Evol. 2012 Mar;2(3):493–500. doi: 10.1002/ece3.94 (PMC3399139; doi:10.1002/ece3.94)
Supplement: Supplementary file 1 [file ece30002-0493-SD1.doc]

Hormiga et al. (2003)

| **Species** | **16S-tRNAleu-ND1** | **18S** | **28S** | **COI** | **H3** | **ITS2** |
| --- | --- | --- | --- | --- | --- | --- |
| *Bolyphantes alticeps* | AY078660 | AY078667 |  | AY078691 | AY078700 |  |
| *Frontinella pyramitela* | AY078661 | AY078672 |  | AY078692 | AY078703 |  |
| *Labulla thoracica* | AY078662 | AY078674 |  | AY078694 | AY078707 |  |
| *Lepthyphantes minutus* | AY078663 | AY078673 |  | AY078689 | AY078705 |  |
| *Linyphia triangularis* | AY078664 | AY078668 |  | AY078693 | AY078702 |  |
| *Microlinyphia dana* | AY078665 | AY078677 |  | AY078690 |  |  |
| *Neriene radiata* | AY078710 | AY078670 |  | AY078696 | AY078709 | AY078774 |
| *Neriene variabilis* | AY078711 | AY078669 |  | AY078699 | AY078706 | AY078775 |
| *Orsonwelles ambersonorum G13* |  |  |  |  |  |  |
| *Orsonwelles ambersonorum G18* |  |  |  |  |  | AY078794 |
| *Orsonwelles arcanus G10* | AY078724 |  |  |  |  | AY078798 |
| *Orsonwelles arcanus G14* | AY078727 |  |  |  |  | AY078799 |
| *Orsonwelles bellum* | AY078740 |  |  |  |  | AY078800 |
| *Orsonwelles calx G3* | AY078718 |  |  |  |  | AY078801 |
| *Orsonwelles calx G40* | AY078738 |  |  |  |  | AY078802 |
| *Orsonwelles calx G41* | AY078736 |  |  |  |  | AY078803 |
| *Orsonwelles falstaffius G7* | AY078723 |  |  | AY078752 |  | AY078784 |
| *Orsonwelles falstaffius G12* | AY078721 |  |  | AY078753 |  | AY078785 |
| *Orsonwelles falstaffius G15* | AY078729 |  |  | AY078754 |  |  |
| *Orsonwelles graphica G1* | AY078728 |  |  | AY078744 |  | AY078776 |
| *Orsonwelles graphica G11* | AY078722 |  |  | AY078745 |  | AY078777 |
| *Orsonwelles iudicium* | AY078739 |  |  | AY078751 |  | AY078783 |
| *Orsonwelles macbeth G42* | AY078734 |  |  | AY078746 |  | AY078778 |
| *Orsonwelles macbeth G45* | AY078735 |  |  | AY078747 |  | AY078779 |
| *Orsonwelles macbeth G46* | AY078714 |  |  | AY078748 |  | AY078780 |
| *Orsonwelles macbeth G55* | AY078712 |  |  | AY078749 |  | AY078781 |
| *Orsonwelles macbeth G56* | AY078713 |  |  | AY078750 |  | AY078782 |
| *Orsonwelles malus G8* | AY078720 |  |  |  |  | AY078797 |
| *Orsonwelles malus G16* | AY078730 | AY078676 | AY078687 | AY078697 | AY078708 | AY078795 |
| *Orsonwelles malus G36* | AY078737 |  |  |  |  | AY078796 |
| *Orsonwelles othello G21* | AY078719 |  |  | AY078757 |  | AY078789 |
| *Orsonwelles othello G39* | AY078742 |  |  | AY078758 |  | AY078790 |
| *Orsonwelles othello G44* | AY078743 |  |  | AY078759 |  | AY078791 |
| *Orsonwelles othello G54* | AY078715 |  |  |  |  | AY078792 |
| *Orsonwelles polites G9* | AY078732 |  |  | AY078756 |  | AY078788 |
| *Orsonwelles polites G19* | AY078726 | AY078671 | AY078686 | AY078698 | AY078701 | AY078786 |
| *Orsonwelles polites G22* | AY078725 |  |  | AY078755 |  | AY078787 |
| *Orsonwelles ventus G4* | AY078717 |  |  |  |  | AY078805 |
| *Orsonwelles ventus G5* | AY078716 |  |  |  |  | AY078806 |
| *Orsonwelles ventus G38* | AY078741 |  |  |  |  | AY078804 |
| *Pityohyphantes costatus* | AY078666 | AY078675 |  | AY078695 |  |  |
| *Tenuiphantes tenebricola* |  |  | AY078688 |  | AY078704 |  |

Jordan et al. (2003)

| **Species** | **Ef1A** | **COII; tRNA-Lys; tRNA-Asxs; (A8); (A6)** |
| --- | --- | --- |
| *Agriocnemis exsudans* | AY179094 | AY179158 |
| *Argia sedula* | AY179095 | AY179159 |
| *Enallagma geminatum* | AY179096 | AY179160 |
| *Ischnura aurora* | AY179097 | AY179161 |
| *Ischnura heterosticta* | AY179098 | AY179162 |
| *Megalagrion adytum A1* | AY179038 | AY179102 |
| *Megalagrion blackburni B33* | AY179042 | AY179106 |
| *Megalagrion calliphya C30* | AY179045 | AY179109 |
| *Megalagrion eudytum E1* | AY179046 | AY179110 |
| *Megalagrion eudytum E8* | AY179047 | AY179111 |
| *Megalagrion hawaiiense Hw1* | AY179048 | AY179112 |
| *Megalagrion hawaiiense Hw2* | AY179050 | AY179114 |
| *Megalagrion hawaiiense Hw81* | AY179051 | AY179115 |
| *Megalagrion hawaiiense Hw59* | AY179052 | AY179116 |
| *Megalagrion heterogamias Ht1* | AY179053 | AY179117 |
| *Megalagrion kauaiense Ka1* | AY179055 | AY179119 |
| *Megalagrion n. sp. SJ-2003 Ka4* | AY179057 | AY179121 |
| *Megalagrion koelense Kl50* | AY179058 | AY179122 |
| *Megalagrion koelense Kl10* | AY179059 | AY179123 |
| *Megalagrion koelense Kl5* | AY179060 | AY179124 |
| *Megalagrion koelense KL94* | AY179061 | AY179125 |
| *Megalagrion koelense Kl6* | AY179062 | AY179126 |
| *Megalagrion leptodemas L1* | AY179064 | AY179128 |
| *Megalagrion mauka Hw40* | AY179066 | AY179130 |
| *Megalagrion mauka M1* | AY179067 | AY179131 |
| *Megalagrion nesiotes Ns3* | AY179068 | AY179132 |
| *Megalagrion nigrohamatum nigrohamatum Nh4* | AY179071 | AY179135 |
| *Megalagrion nigrohamatum nigrolineatum Nl3* | AY179072 | AY179136 |
| *Megalagrion oahuense Oa4* | AY179073 | AY179137 |
| *Megalagrion oceanicum Oc1* | AY179075 | AY179139 |
| *Megalagrion oresitrophum Op9* | AY179078 | AY179142 |
| *Megalagrion orobates Ob1* | AY179079 | AY179143 |
| *Megalagrion orobates OB3* | AY179081 | AY179145 |
| *Megalagrion pacificum Pc8* | AY179082 | AY179146 |
| *Megalagrion pacificum Pc21* | AY179084 | AY179148 |
| *Megalagrion paludicola Pa1* | AY179085 | AY179149 |
| *Megalagrion vagabundum V1* | AY179087 | AY179151 |
| *Megalagrion xanthomelas X74* | AY179092 | AY179156 |
| *Pseudagrion civicum* | AY179099 | AY179163 |
| *Pseudagrion pacificum* | AY179100 | AY179164 |
| *Pseudagrion palauense* | AY179101 | AY179165 |

Givnish et al. (2008)

| **Species** | **psbA-trnH** | **rpl16 intron** | **trnL-trnF** | **trnT-trnL** | **trnV-trnK** | **atpB-rbcL** |
| --- | --- | --- | --- | --- | --- | --- |
| *Brighamia insignis* | DQ272693 | DQ285100 | DQ285139 | DQ285217 | DQ285178 | DQ285256 |
| *Brighamia rockii* | DQ272694 | DQ285101 | DQ285140 | DQ285218 | DQ285179 | DQ285257 |
| *Clermontia arborescens* | DQ272695 | DQ285102 | DQ285141 | DQ285219 | DQ285180 | DQ285258 |
| *Clermontia fauriei* | DQ272696 | DQ285103 | DQ285142 | DQ285220 | DQ285181 | DQ285259 |
| *Clermontia kakeana* | DQ272697 | DQ285104 | DQ285143 | DQ285221 | DQ285182 | DQ285260 |
| *Clermontia parviflora* | DQ272725 | DQ285132 | DQ285171 | DQ285249 | DQ285210 | DQ285288 |
| *Cyanea acuminata* | DQ272698 | DQ285105 | DQ285144 | DQ285222 | DQ285183 | DQ285261 |
| *Cyanea coriacea* | DQ272699 | DQ285106 | DQ285145 | DQ285223 | DQ285184 | DQ285262 |
| *Cyanea floribunda* | DQ272727 | DQ285134 | DQ285173 | DQ285251 | DQ285212 | DQ285290 |
| *Cyanea hirtella* | DQ272729 | DQ285136 | DQ285175 | DQ285253 | DQ285214 | DQ285292 |
| *Cyanea kuhihewa* | DQ272731 | DQ285138 | DQ285177 | DQ285255 | DQ285216 | DQ285294 |
| *Cyanea leptostegia* | DQ272726 | DQ285133 | DQ285172 | DQ285250 | DQ285211 | DQ285289 |
| *Cyanea pilosa subsp. Longipedunculata* | DQ272728 | DQ285135 | DQ285174 | DQ285252 | DQ285213 | DQ285291 |
| *Delissea rhytidosperma* | DQ272700 | DQ285107 | DQ285146 | DQ285224 | DQ285185 | DQ285263 |
| *Delissea subcordata* | DQ272701 | DQ285108 | DQ285147 | DQ285225 | DQ285186 | DQ285264 |
| *Lobelia gloria-montis* | DQ272702 | DQ285109 | DQ285148 | DQ285226 | DQ285187 | DQ285265 |
| *Lobelia hypoleuca* | DQ272703 | DQ285110 | DQ285149 | DQ285227 | DQ285188 | DQ285266 |
| *Hillebr. Lobelia kauaensis* | DQ272704 | DQ285111 | DQ285150 | DQ285228 | DQ285189 | DQ285267 |
| *Lobelia niihauensis* | DQ272705 | DQ285112 | DQ285151 | DQ285229 | DQ285190 | DQ285268 |
| *Lobelia villosa* | DQ272730 | DQ285137 | DQ285176 | DQ285254 | DQ285215 | DQ285293 |
| *Lobelia yuccoides* | DQ272706 | DQ285113 | DQ285152 | DQ285230 | DQ285191 | DQ285269 |
| *Trematolobelia kauaiensis* | DQ272707 | DQ285114 | DQ285153 | DQ285231 | DQ285192 | DQ285270 |
| *Trematolobelia macrostachys* | DQ272708 | DQ285115 | DQ285154 | DQ285232 | DQ285193 | DQ285271 |
| *Apetahia longistigmata* | DQ272709 | DQ285116 | DQ285155 | DQ285233 | DQ285194 | DQ285272 |
| *Apetahia margaretae* | DQ272723 | DQ285130 | DQ285169 | DQ285247 | DQ285208 | DQ285286 |
| *Burmeistera crispiloba* | DQ272718 | DQ285125 | DQ285164 | DQ285242 | DQ285203 | DQ285281 |
| *Centropogon gutierrezii* | DQ272719 | DQ285126 | DQ285165 | DQ285243 | DQ285204 | DQ285282 |
| *Isotoma axillaris* | DQ272720 | DQ285127 | DQ285166 | DQ285244 | DQ285205 | DQ285283 |
| *Lobelia boninensis* | DQ272711 | DQ285118 | DQ285157 | DQ285235 | DQ285196 | DQ285274 |
| *Lobelia cardinalis* | DQ272722 | DQ285129 | DQ285168 | DQ285246 | DQ285207 | DQ285285 |
| *Lobelia columnaris* | DQ272712 | DQ285119 | DQ285158 | DQ285236 | DQ285197 | DQ285275 |
| *Lobelia excelsa* | DQ272713 | DQ285120 | DQ285159 | DQ285237 | DQ285198 | DQ285276 |
| *Lobelia giberroa* | DQ272714 | DQ285121 | DQ285160 | DQ285238 | DQ285199 | DQ285277 |
| *Lobelia nicotianaefolia* | DQ272715 | DQ285122 | DQ285161 | DQ285239 | DQ285200 | DQ285278 |
| *Lobelia organensis* | DQ272716 | DQ285123 | DQ285162 | DQ285240 | DQ285201 | DQ285279 |
| *Lobelia petiolata* | DQ272717 | DQ285124 | DQ285163 | DQ285241 | DQ285202 | DQ285280 |
| *Lobelia vivaldii* | DQ272721 | DQ285128 | DQ285167 | DQ285245 | DQ285206 | DQ285284 |
| *Sclerotheca forsteri* | DQ272724 | DQ285131 | DQ285170 | DQ285248 | DQ285209 | DQ285287 |
| *Sclerotheca jayorum* | DQ272710 | DQ285117 | DQ285156 | DQ285234 | DQ285195 | DQ285273 |
